# Chronic pain, mental health and functional impairment in adult refugees from Syria resettled in Norway: a cross-sectional study

**DOI:** 10.1186/s12888-022-04200-x

**Published:** 2022-08-24

**Authors:** Alexander Nissen, Kamila Angelika Hynek, David Scales, Per Kristian Hilden, Melanie Straiton

**Affiliations:** 1 Section for Trauma, Catastrophes and Forced Migration - Adults and Elderly, Norwegian Centre for Violence and Traumatic Stress Studies, 0484 Oslo, Norway; 2 Division for Mental and Physical Health, Norwegian Institute of Public Health, Skøyen, PO Box 222, 0213 Oslo, Norway; 3 Section of Hospital Medicine, Division of General Internal Medicine, Department of Internal Medicine, Weill Cornell Medicine, 525 E 68th Street, Box 331, New York, NY 10065 USA

**Keywords:** Refugees, Syria, Mental health, Pain, Anxiety, Depression, PTSD, Functional impairment

## Abstract

**Background:**

Limited research exists on pain and especially the co-occurrence of pain and mental ill health in general refugee populations. The present study aimed to approximate the prevalence of chronic pain (CP) among adult refugees from Syria resettled in Norway; investigate the association between CP and mental ill health; and explore how CP and mental ill health associate with both perceived general health and functional impairment. Gender as potential effect modifier in these associations was also examined.

**Methods:**

Cross-sectional, postal survey questionnaire. Inclusion criteria: ≥ 18 years old; refugee from Syria; and arrived in Norway between 2015 and 2017. Study sample was randomly drawn from full population registries, and *n* = 902 participated (participation rate ≈10%). CP was measured with 10 items on pain lasting for ≥ 3 consecutive months last year. Symptoms of anxiety, depression and PTSD were measured with the HSCL and HTQ scales, respectively. Ordered and binomial logistic regressions were used in analyses. Gender was tested as effect modifier with Wald test for interaction.

**Results:**

In the sample overall, the proportion of participants who reported severe CP was 43.1%. There was strong evidence that anxiety, depression and PTSD were associated with higher levels of CP. In fully adjusted regression models, including both CP and mental health variables, CP was strongly associated with poor perceived general health whereas mental health showed much weaker associations. The association between mental health (anxiety and PTSD) and functional impairment was highly gender specific, with strong associations in men but not in women. CP was strongly associated with functional impairment with no difference across gender.

**Conclusion:**

The study shows a high burden of CP in a general population of adult refugees from Syria with likely substantial adverse consequences for daily functioning**.** The strong association between CP and mental ill health suggests personnel working with refugees’ health should be attuned to their co-occurrence as both problems may need to be addressed for either to be effectively mitigated. A clear mismatch exists between the burden on health caused by pain in general refugee populations and the amount of available evidence to guide mitigating strategies.

**Trial registration:**

NCT03742128.

**Supplementary Information:**

The online version contains supplementary material available at 10.1186/s12888-022-04200-x.

## Background

A substantial body of evidence across various settings and populations has documented the close interconnectedness of psychological distress and somatic symptoms in general [1–3], and pain in particular [4–7]. The underlying mechanisms behind the frequent co-occurrence of psychological distress and pain are complex, likely bidirectional and involve both genetic and environmental factors [7, 8]. For traumatized individuals with post-traumatic stress symptoms, the experience of elevated levels of pain is thought to be linked to disturbances in the processing of pain stimuli (pain sensitization); hypermnesia or overconsolidation of traumatic memories (imprinting); and synergistic physiological effects between trauma-related anxiety and pain perception. Transcultural psychiatric approaches stress the importance of sociocultural elements when investigating psychological and somatic symptoms across populations and the need to understand local illness models and idioms of distress [9–11]. Within this framework, variations in patterns of co-occurrence across ethnocultural groups documented in some studies are at least partly explained by cultural differences in how distress is experienced, represented and expressed, and relatedly, by fear of stigmatization [12–14].

Refugees are often exposed to a number of potentially traumatic experiences both before and during flight, and many experience post-migration hardships after resettling in a new country. The cumulative load of these stressors increases refugees’ risk of mental ill health [15–18]. Elevated rates of post-traumatic stress disorder (PTSD), depression and anxiety have repeatedly been found in refugee populations (e.g., [19, 20]). High levels of pain have also been documented in studies on selected refugee groups – e.g. war-wounded refugees, refugees exposed to torture and refugees in clinical settings [21–26]. However, studies on pain in general refugee populations are scant. One recent, two-wave, longitudinal study on resettled Syrian refugees in Norway found chronic pain to be fairly constant over time at around 30% [27], and pain was found to be frequently reported among refugees from Syria residing in Turkey [28] and among Rohingya refugees in Bangladesh [29].

In a work commissioned by the UNHCR on culture, context and the mental health and psychosocial well-being of Syrians from 2015, Hassan et al. note that “…most Arabic and Syrian idioms of distress do not separate somatic experience and psychological symptoms, because body and soul are interlinked in explanatory models of illness” (p. 22 in [30]). They further note potential stigma against expressing certain negative emotions, especially in men and if emotions are labeled as ‘psychological’ or ‘psychiatric’. The issue of stigma against negative emotions is also pointed out by other researchers working with Arab populations [12]. As a consequence, it has been noted that physical symptoms may be the initial and predominant presenting complaints for people with psychological or mental health issues [30]. With several studies documenting a high burden of mental ill health among refugees from Syria in recent years (for an overview, please refer to [31]), it seems vital, therefore, to expand knowledge on the co-occurrence and possible relationship of psychological and somatic symptoms in this population. This could be of value to healthcare providers by sensitizing them to the complex manifestations of distress, which in turn could help ensure needs are recognized and addressed adequately. Yet, there is notable shortage of studies addressing this topic, both among refugees from Syria and among other refugee populations.

The present study focuses on pain and the co-occurrence of pain and psychological distress in a general refugee population. Nonetheless, due to the limited number of studies on this topic, relevant evidence from a broader range of somatic symptoms, including the category formerly known as “somatoform disorders” in the DSM diagnostic systems, is at times included. Pain is both a central feature of most instruments used to measure somatic distress/somatization and one of the most frequently reported somatic symptoms in refugee populations (e.g., [28, 32]). Moreover, evidence suggests symptom groups (e.g. musculoskeletal, gastrointestinal) do not show differential association with psychological distress [1]. Importantly, the “somatoform disorders” have been reconceptualized in the most recent versions of both major diagnostic frameworks (DSM-V and ICD-11), partly to remove “medically unexplained symptoms” as a diagnostic criterion, which was thought to be unreliable, stigmatizing to patients and ‘perilous in its reliance on mind–body dualism’ (p. 233 in [33]). The latter point may be particularly relevant in the context of illness models with a less dichotomous understanding of body and mind. Therefore, evidence from studies on somatic distress/somatization in refugee populations should be interpreted with care. Interpretations must incorporate an understanding of and appreciation for local idioms and explanatory models of distress, and, even if no medical explanation is found for somatic symptoms, that does not mean that none exists [34]. For example, many refugees have been exposed to torture which can have long-term consequences for, and a complex relationship with, pain [35, 36].

We have only identified one study exploring psychological distress and pain in a general refugee population, which was conducted on Syrian refugees resettled in Norway [37]. Interestingly, the study found no associations between pain and mental ill health (i.e. anxiety, depression and PTSD) in the first wave of the study, when refugees were still residing in Lebanon, awaiting resettlement. However, fairly strong associations were found at the follow-up measurement about a year after resettlement. The latter finding is broadly supported by three recent and large cross-sectional studies on refugees and war-displaced persons which found clear associations between anxiety, depression and PTSD and somatic distress, as measured by the Patient Health Questionnaire (PHQ-15), where pain is a core component [28, 38, 39]. Further support comes from a matched control study on Bhutanese torture survivors, which found that somatic complaints were associated with PTSD and depression [40], and from studies on clinical subsamples documenting close links between pain and mental ill health [24, 41–43].

The associations of pain with functional impairment and perceived general health are also understudied in refugee populations. The degree of pain experienced by severely traumatized and tortured refugees and the associated level of functional disability has been shown to be substantial compared to many non-refugee groups suffering from pain conditions [43, 44]. Pain has also been strongly linked to low health-related wellbeing among female Yazidi refugees who experienced extreme violence by the “Islamic state” [32]. Furthermore, in a general population of conflict-affected persons in the Republic of Georgia, somatic distress was strongly associated with increased functional disability, even after controlling for mental ill health [38]. We know of no prior study that has investigated functional impairment in relation to pain and mental ill health (i.e. anxiety, depression and PTSD) in refugees.

An abundance of evidence from both refugee and non-refugee populations has documented that pain conditions and somatic distress are substantially more prevalent in women (e.g., [28, 38, 39, 45, 46]), suggesting studies on this topic should investigate and report gender-specific patterns. This aligns with general recommendations for research on refugees which emphasize the importance of exploring the role of gender across studies, as this has been insufficiently prioritized in the past [20, 47]. Recent evidence suggests gender is a key moderating factor between various migration-related stressors and mental health [48–50].

Using cross-sectional questionnaire data from adult refugees from Syria resettled in Norway, the primary aims of the present study were to assess the burden of chronic pain in this population and to explore the associations of chronic pain with anxiety, depression and PTSD. Secondary aims included to investigate unique and overlapping associations of chronic pain and mental health with perceived general health and functional impairment, and to test gender as an effect modifier in the above associations.

## Methods

The primary aims were broadly outlined in the study’s pre-registration in ClinicalTrial.gov (NCT03742128, 15/11/2018), though some data-handling decisions were made with data in-house and deviated slightly from the registered plan (these are highlighted in Additional file 1, which also includes the pre-registered analysis plans). All regression models were built with data at hand, and secondary aims were developed post-hoc, meaning these are hypothesis-generating rather than hypothesis-testing even if they are rooted in theory and prior research [51, 52].

### Design and participants

The present study used a questionnaire-based cross-sectional design. Eligible participants included all adult (≥ 18 years old) refugees from Syria who were granted temporary or permanent residency in Norway between 2015 and 2017. A complete list of eligible participants was obtained from the population registry in Norway (*N* = 14,350), and from this list, 10,000 individuals were randomly sampled for participation. All sampled participants were sent a study invitation letter together with the actual questionnaire and an informed consent form to be signed and returned with the questionnaire, all in formal Arabic. The letter also contained contact information in the event of adverse reactions following participation. The invitations were sent out in November 2018. Due to a low response rate, the study did not close until September 2019. Several efforts were made to boost participation including one postal or telephone reminder to all non-responders. The Regional Committees for Medical and Health Research Ethics (REK) – Region Southeast (A) in Norway granted ethical approval for the study (reference number 2017/1252). All parts of the study were conducted in accordance with the protocol approved by REK and in line with relevant regulations.

### Variables and measurements

#### Chronic pain (CP)

Questions on CP consisted of two parts. The first part concerned musculoskeletal pain- and stiffness and was taken from the Tromsø Study which has been used extensively in the last decades in many large epidemiological studies in Norway (e.g., [53, 54]). Specifically, participants were asked to indicate whether they had been troubled with pain or stiffness in muscle and joints for a minimum of three consecutive months in the last year. The question was asked for five different body regions (neck/shoulders; arms/hands; upper back; lower back; hips/legs/feet), all with the same three answer choices provided: not troubled (= 1); somewhat troubled (= 2); very troubled (= 3). In the second part, participants were asked to indicate whether they had been troubled with pain for more than three consecutive months in the last year in five other body regions (stomach; head; genital area; chest; other), using the same answer choices. These items were also based on the Tromsø study, though slightly adapted. For each of the two parts, participants who answered 1 (not troubled) on all items and had a maximum of one missing, were categorized as “no CP”. Participants who answered at least one item with 2 (somewhat troubled) or 3 (very troubled), were categorized as “some CP” or “severe CP”, respectively, regardless of the number of missing [53]. The two parts were then combined for an overall CP variable with 3 categories coded as follows: “No CP” = “not troubled” for both parts; “Some CP” = at least one part with “somewhat troubled” (and no part with “very troubled”); and “Severe CP” = at least one part with “very troubled”.

#### Perceived general health (PGH)

Question taken from the European Social Survey 2010 [55]. The question asks “How is your health in general” and provides six answer choices: very bad (= 1); bad (= 2); fair (= 3); good (= 4); very good (= 5) and don’t know (= 6). The variable was dichotomized into “Good PGH” (answer choices 4 and 5) and “Poor PGH” (answer choices 1–3). The “don’t know” category was recoded as missing.

#### Functional impairment (FI)

Question taken from the European Social Survey 2010 [56]. The question asks: “Are you hampered in your daily activities in any way by any longstanding illness, or disability, infirmity or mental health problem?”. There are four possible answer choices provided: no (= 1); yes, to some extent (= 2); yes, a lot (= 3); and don’t know (= 4). This variable was dichotomized into “No FI” (answer choice 1) vs. “Some/a lot FI” (answer choices 2 and 3), whereas the “don’t know” category was recoded as missing.

#### Mental ill health

Symptoms of anxiety and depression were measured with the 25 item Hopkins Symptom Checklist, HSCL-25 [57], whereas PTSD symptoms were measured by the Harvard Trauma Questionnaire (HTQ) [58]. The first ten items of HSCL-25 measure anxiety and the last 15 depression. All items are scored on a 4-point scale from 1 (“not at all”) to 4 (“extremely”). A mean-item score was estimated for participants with < 3 missing on each subscale, and two dichotomized variables, HSCL-anxiety and HSCL-depression, were created based on cut-offs of 1.75 and 1.80 for anxiety and depression, respectively. The choice of cut-off was made to be consistent with the sister-study in Sweden [59], and was pre-registered prior to study start. It was also the chosen cut-off in a prior publication assessing the burden of mental ill health in the present study sample [60]. The 16 items used from the HTQ (section IV) are also scored from 1 (“not at all”) to 4 (“very much”), and a mean-item score was created for those with < 3 missing. A dichotomized HTQ-PTSD variable was created to indicate those with screen-positive PTSD with a cut-off of 2.06, using the same rationale for the choice of cut-off as above. Both scales have been widely used to measure mental health problems in refugees, and several studies have supported the scales’ validity and reliability across different refugee populations (e.g., [61–63]), though recent evidence suggests caution may be warranted before using the depression subscale in Arabic speaking refugees [64]. Cronbach’s alpha for the scales in the present sample were 0.96 (HSCL_total_), 0.93 (HSCL_anxiety_), 0.94 (HSCL_depression_), and 0.92 (HTQ_total_).

#### Potentially traumatic experiences (PTEs)

PTEs prior to- and during flight was measured with the Refugee Trauma History Checklist (RTHC), created and validated by Sigvardsdotter and colleagues [65, 66]. The scale asks about eight PTEs prior to flight (e.g. “War at close quarter”, “Torture”) and the same PTEs during flight for a total of 16 items. Each item is answered with Yes/No. A new variable named PTE-adversity ratio (PTE-AR) was created based on the work by Steel et al. [15], which equaled the number of endorsed items (i.e. “Yes”) divided by 16. The variable was categorized into four groups: < 0.2; 0.2–0.29; 0.3–0.39; and ≥ 0.4.

#### Sociodemographic and background variables

Age and gender were obtained from Norwegian registry data, with age categorized into 18–29 years; 30–39 years; 40–49 years; 50–64 years; and ≥ 65 years in line with the study’s pre-registration. Self-reported data was used for marital status (categorized into: unmarried; married/registered partner; and divorced/separated/widow(er), years of education (categorized into: ≤ 9 years; 10–12 years; and > 12 years), refugee status (asylum seeker; quota refugee; and family reunion), and immigration year (categorized into: 2010–2015; 2016; and 2017).

The items used to measure CP, PGH, FI and sociodemographic/background variables were translated into Arabic using a professional translation bureau. The translation was subsequently checked by two independent translators and discussed with an Arabic speaking reference group from Syria residing in Norway. The translations of the HSCL, HTQ and RTHC scales were obtained from collaborators at the Swedish Red Cross University College in Sweden, who used double-blind and back-translation procedures in addition to interviews with Arabic-speakers to ensure validity [59].

### Statistical analysis

Data was first inspected for errors, outliers, missing and distributional properties. Frequency distributions and cross tabulations with chi-square test of equal proportions were used to make descriptive tables and investigate bivariate associations. Ordered and regular logistic regression models were used to investigate the secondary aims of the study. All regression models were built in a forward manner, with sociodemographic/background variables only included in adjusted models if they were associated with the respective outcomes in bivariate analyses (*p*-value < 0.05). Gender, age and PTE-AR were included in adjusted models for a priori reasons regardless of bivariate association given their known association with both pain and mental health. The PTE-AR categories 0.20–0.29 and 0.30–0.39 and the immigration year categories 2016 and 2017 were combined in regression analysis due to few observations in the individual categories. Due to a fairly high number with missing data across variables, the regression analyses were done on imputed data. The imputed values were obtained using multiple imputation by chained equations, MICE [67], with all variables included in the imputation model.

Crude and adjusted ordered logistic regressions were used to investigate the associations of anxiety, depression and PTSD with CP. The odds ratios (ORs) presented indicate the odds of being in the higher category of CP for different predictor levels, regardless of which of the two possible ways CP is dichotomized – i.e. (3 and 2) vs. 1 or 3 vs. (2 and 1). Crude and adjusted logistic regressions were used to investigate the association of mental health variables and CP with perceived general health and functional impairment. Spearman’s rank correlation was used to investigate possible multicollinearity between mental health variables and CP (deemed problematic if > 0.7). The Wald test for interaction was used to test whether gender modified the associations of mental ill health with CP, and/or the associations of mental ill health and CP with perceived general health and functional impairment.

#### Sensitivity analyses

Two sensitivity analyses were done on the fully adjusted regression models. First, all models were repeated with the chronic pain variable split into its two constituent parts: pain/stiffness in muscles and joints (5 items), and pain in other body part (5 items). Furthermore, given the abovementioned evidence questioning the construct validity of the HSCL among certain refugee populations [64], and the known theoretical and empirical overlap between anxiety, depression and PTSD, regression models were repeated with anxiety, depression and PTSD combined into *one* common-mental-health-problem variable. This dichotomous variable, “Any mental health problem”, was coded as “Yes” (= 1) if *any* of anxiety, depression or PTSD were above threshold, and No (= 0) if *all* of them were below threshold. The sensitivity analyses are presented in Additional files 2 and 3. Additional file 2 also contains detailed distributions of the two constituent parts of chronic pain.

Analyses were performed with Stata version 17 (StataCorp. 2021. Stata Statistical Software: Release 17. College Station, TX: StataCorp LLC).

## Results

Of the roughly 10,000 invitations for participation sent out, 1,235 were returned due to incorrect address. Of those who presumably received the invitation, 902 participated, giving a response rate of about 10%. Participants were older and a higher proportion were married compared to the sample population, though there was no difference in gender distribution. For further statistics relating to representativity and possible selection bias problems, we refer to a prior publication [68]. Overall, 64.5% of participants were men, and the mean age was 38.9 (SD = 11.6; min. age = 19; max. age = 83). The degree of exposure to PTEs was high with over 50% in the highest category (affirmative on ≥ 40% of individual PTEs inquired about). Men reported more PTEs than women (Chi-square test of equal PTE-AR across gender: *p*-value = 0.01). The unweighted approximated prevalence of anxiety, depression and PTSD based on screening instruments were 31.7%, 40.0% and 34.7%, respectively, with no statistically significant difference between genders at the *p* = 0.05 threshold. Table 1 gives further descriptive statistics for all participants and stratified by gender.

**Table 1:** Descriptive statistics, all respondents and stratified by gender (*n* = 902)

		All respondents		Stratified by gender			
				Men (*n* = 582)		Women (*n* = 320)	
		n	(%)	n	(%)	n	(%)
Age (*n* = 902)	18–29 yrs	197	( 21.8)	119	(20.5)	78	(24.4)
	30–39 yrs	310	( 34.4)	205	(35.2)	105	(32.8)
	40–49 yrs	230	( 25.5)	144	(24.7)	86	(26.9)
	≥ 50	165	( 18.3)	114	(19.6)	51	(15.9)
Education (*n* = 882)	≤ 9 yrs	394	(44.7)	244	(43.0)	150	(47.8)
	10–12 yrs	158	(17.9)	91	(16.0)	67	(21.3)
	> 12 yrs	330	(37.4)	233	(41.0)	97	(30.9)
Marital status (*n* = 902)	Unmarried	236	(26.1)	199	(34.2)	37	(11.6)
	Married/partner	595	(66.0)	349	(60.0)	246	(76.9)
	Divorced/wid	71	(7.9)	34	(5.8)	37	(11.6)
Refugee status (*n* = 860)	Asylum seeker	454	(52.8)	366	(65.8)	88	(29.0)
	Quota refugee	273	(31.7)	154	(27.7)	119	(39.1)
	Family reunion	133	(15.5)	36	(6.5)	97	(31.9)
Immigration year (*n* = 856)	2010–2015	509	(59.5)	394	(71.4)	115	(37.8)
	2016	163	(19.0)	72	(13.0)	91	(29.9)
	2017	184	(21.5)	86	(15.6)	98	(32.2)
PTE-AR (*n* = 819)	< 0.20	163	(19.9)	92	(17.4)	71	(24.5)
	0.20–0.29	79	(9.7)	44	(8.3)	35	(12.1)
	0.30–0.39	158	(19.3)	103	(19.5)	55	(19.0)
	≥ 0.40	419	(51.1)	290	(54.8)	129	(44.4)
HSCL-anxiety^a^ (*n* = 886)	No	605	(68.3)	403	(70.3)	202	(64.5)
	Yes	281	(31.7)	170	(29.7)	111	(35.5)
HSCL-depression^b^ (*n* = 877)	No	526	(60.0)	344	(60.3)	182	(59.5)
	Yes	351	(40.0)	227	(39.7)	124	(40.5)
HTQ-PTSD^c^ (*n* = 877)	No	573	(65.3)	361	(64.0)	212	(67.7)
	Yes	304	(34.7)	203	(36.0)	101	(32.3)

*PTE-AR* Potentially Traumatic Experiences-Adversity Ratio

^a ^Mean-item score on HSCL anxiety subscale > 1.75

^b ^Mean-item score on HSCL depression subscale > 1.80

^c ^Mean-item score on HTQ ≥ 2.06

Table 2 shows the frequency distributions of CP, PGH and FI, and bivariate associations with predictors/covariates of interest. For the total sample, 43.1% reported severe CP, 39.9% poor PGH and 34.9% at least some degree of FI. Being a woman was positively associated with CP and poor PGH, but not FI. There was very strong evidence (*p*-value < 0.001) that anxiety, depression and PTSD were associated with elevated levels of CP, poor PGH and FI in bivariate analyses.

**Table 2:** Frequency distributions of chronic pain^a^, perceived overall health and impaired daily functioning due to longstanding illness

		Chronic pain^a^ (%)				Perceived general health (%)			Impaired functioning (%)		
		No	Some	Severe	χ2	Very good/good	Fair/poor/very poor	χ2	No	Some/a lot	χ2
Total sample		19.8	37.1	43.1		60.1	39.9		65.1	34.9	
Gender	Men	22.3	37.5	40.2	0.013	63.0	37.0	0.022	65.8	34.2	0.605
	Women	15.2	36.2	48.6		54.9	45.1		64.0	36.0	
Age	18–29 yrs	31.3	37.4	31.3	< 0.001	77.9	22.1	< 0.001	80.6	19.4	< 0.001
	30–39 yrs	22.6	39.5	37.9		67.5	32.5		70.4	29.6	
	40–49 yrs	15.5	37.2	47.3		56.4	43.6		65.4	34.7	
	≥ 50	6.8	31.7	61.5		30.4	69.6		39.1	60.9	
Education	≤ 9 yrs	15.5	37.5	47.0	0.061	54.3	45.7	0.007	60.1	39.9	0.028
	10–12 yrs	22.6	36.8	40.6		61.9	38.1		67.7	32.3	
	> 12 yrs	23.3	37.1	39.6		65.8	34.2		70.0	30.0	
Marital status	Unmarried	28.2	35.9	35.9	< 0.001	70.6	29.4	< 0.001	70.9	29.1	0.005
	Married/partner	18.6	37.9	43.5		58.4	41.6		65.0	35.0	
	Divorced/widowed	2.9	33.3	63.8		38.7	61.3		48.4	51.6	
Refugee status	Asylum seeker	21.6	39.1	39.3	0.211	61.5	38.5	0.806	69.7	30.3	0.098
	Quota refugee	16.7	35.2	48.1		59.2	40.8		61.5	38.5	
	Family reunion	21.1	36.0	42.9		61.9	38.1		67.8	32.2	
Year immigration	2010–2015	20.9	37.3	41.8	0.024	61.6	38.4	0.042	66.8	33.2	0.103
	2016	16.8	28.5	54.7		51.3	48.7		57.8	42.2	
	2017	19.6	41.9	38.5		63.5	36.5		67.9	32.1	
PTE-AR	< 0.20	30.2	39.0	30.8	< 0.001	70.9	29.1	< 0.001	79.9	20.1	< 0.001
	0.20–0.29	25.6	37.2	37.2		70.1	29.9		73.0	27.0	
	0.30–0.39	20.5	40.4	39.1		62.8	37.3		67.7	32.4	
	≥ 0.40	14.9	34.7	50.4		51.9	48.1		55.8	44.2	
HSCL^b^-anxiety	No	26.8	43.0	30.2	< 0.001	71.9	28.1	< 0.001	78.7	21.3	< 0.001
	Yes	5.4	25.1	69.5		34.6	65.4		35.5	64.5	
HSCL^c^-depression	No	28.1	45.1	26.8	< 0.001	75.5	24.5	< 0.001	81.2	18.8	< 0.001
	Yes	8.0	25.2	66.8		36.7	63.3		40.0	60.0	
HTQ^d^-PTSD	No	27.2	43.5	29.3	< 0.001	73.2	26.8	< 0.001	79.7	20.3	< 0.001
	Yes	6.3	25.6	68.1		34.1	65.9		36.3	63.7	

*PTE-AR* Potentially Traumatic Experiences-Adversity Ratio

^a^ Chronic pain (CP) was measured through 10 items answered on a 3-point Likert scale (no/somewhat/very troubled) and categorized into 3 groups: no CP (“no” on all 10 items); some CP (“somewhat troubled” on at least one item and no item as “very troubled”); and severe CP (“very troubled” on at least one item). All items asked about pain lasting for at least 3 months in the last year

^b^ Mean-item score on HSCL anxiety subscale > 1.75

^c^ Mean-item score on HSCL depression subscale > 1.80

^d^ Mean-item score on HTQ ≥ 2.06

Table 3 presents crude and adjusted ordered logistic regression models of CP. Being a woman was associated with roughly 50% higher odds of CP in both unadjusted and adjusted models (OR_fully adjusted_ = 1.55; 95% CI 1.13–2.12). There was very strong evidence in both crude and adjusted models that all mental ill health variables (i.e. anxiety, depression and PTSD) were associated with increased odds of CP (anxiety: OR_fully adjusted_ = 2.42, 95% CI 1.64–3.55; depression: OR_fully adjusted_ = 2.28, 95% CI 1.53–3.42; and PTSD: OR_fully adjusted_ = 1.97, 95% CI 1.32–2.94). Wald test for interaction between gender and each of mental ill health variables did not reach statistical significance, though there was a tendency for a stronger association of anxiety with CP in women (*p*-value = 0.085). Furthermore, there was very strong evidence (*p* < 0.001) that older age was associated with higher levels of CP, and some evidence that marital status (i.e. widow(er)/separated/divorced vs. unmarried, *p* = 0.021) was associated with higher levels of CP.

**Table 3:** Crude and adjusted ordered logistic regression models of chronic pain^a^

		Unadjusted		Partially adjusted^d^						Fully adjusted		
		Model 1		Model 2^i^		Model 2^ii^		Model 2^iii^		Model 3		
		uOR	95% CI	aOR	95% CI	aOR	95% CI	aOR	95% CI	aOR	95% CI	Wald^b^
Gender^b^	Women	1.46^**^	(1.13–1.89)	1.57^**^	(1.15–2.14)	1.55^**^	(1.14–2.12)	1.79^***^	(1.31–2.43)	1.55^**^	(1.13–2.12)	
HSCL-Anxiety^b^	Yes	5.45^***^	(4.04–7.35)	5.17^***^	(3.76–7.11)					2.42^***^	(1.64–3.55)	0.085
HSCL-Dep^b^	Yes	5.46^***^	(4.12–7.23)			5.30^***^	(3.91–7.18)			2.28^***^	(1.53–3.42)	0.134
HTQ-PTSD^b^	Yes	5.09^***^	(3.81–6.79)					4.90^***^	(3.57–6.73)	1.97^**^	(1.32–2.94)	0.536
Age	30–39 years	1.47^*^	(1.05–2.05)	1.62^*^	(1.11–2.37)	1.51^*^	(1.03–2.22)	1.57^*^	(1.07–2.30)	1.56^*^	(1.06–2.29)	
	40–49 years	2.19^***^	(1.52–3.14)	2.53^***^	(1.64–3.92)	2.24^***^	(1.45–3.46)	2.28^***^	(1.47–3.53)	2.36^***^	(1.52–3.67)	
	≥ 50 years	3.90^***^	(2.60–5.85)	3.90^***^	(2.37–6.41)	3.61^***^	(2.20–5.94)	3.77^***^	(2.30–6.19)	3.58^***^	(2.17–5.91)	
Marital status	Married/partner	1.49^**^	(1.12–1.97)	0.97	(0.67–1.40)	1.07	(0.74–1.56)	0.98	(0.67–1.42)	1.13	(0.78–1.65)	
	Prev. married^c^	3.73^***^	(2.19–6.36)	1.92^*^	(1.03–3.59)	1.90^*^	(1.02–3.56)	1.92^*^	(1.03–3.58)	2.12^*^	(1.12–3.99)	
Immigration year	2016/2017	1.17	(0.90–1.50)	1.06	(0.79–1.42)	1.18	(0.88–1.59)	1.06	(0.79–1.42)	1.17	(0.86–1.58)	
PTE-AR	0.20–0.39	1.44	(1.00–2.07)	1.34	(0.92–1.95)	1.33	(0.91–1.94)	1.21	(0.83–1.76)	1.21	(0.83–1.78)	
	≥ 0.40	2.41^***^	(1.72–3.38)	1.65^**^	(1.15–2.36)	1.52^*^	(1.06–2.19)	1.41	(0.98–2.03)	1.26	(0.87–1.82)	

Reference groups: men (gender); no (mental health variables); 18–29 years (age); unmarried (marital status); 2010–2015 (Immigration. year); < 0.20 (PTE-AR)

^a^ Chronic pain (CP) was measured through 10 items answered on a 3-point Likert scale (no/somewhat/very troubled) and categorized into 3 groups: no CP (“no” on all 10 items); some CP (“somewhat troubled” on at least one item and no item as “very troubled”); and severe CP (“very troubled” on at least one item)

^b^ Interaction between gender and each of the three mental health variables was tested using Wald test of H_0_ = no interaction (ns = *p* > 0.10). Interactions were tested sequentially – i.e., only one interaction term was tested at a time

^c^ Category included: divorced/separated/widow(er)

^d^ In order to evaluate the association between each individual measure of mental ill health and CP adjusted for background variables and PTE only (but not the other two mental ill health variables), three separate partially adjusted models were run (i.e. Models 2^i^, 2^ii^ and 2^iii^)

^*^p < 0.05

^**^p < 0.01

^***^p < 0.001

The mean score for perceived general health (PGH) was 3.614 (SD = 1.063). Table 4 summarizes logistic regression models of PGH (poor = 1 vs. reference group good = 0), focusing on the predictors gender, mental ill health and CP. Gender was not associated with PGH in fully adjusted models at the *p* = 0.05 level, though there was weak evidence in unadjusted and partially adjusted models. Anxiety, depression and PTSD were all strongly associated with PGH in crude models and fairly strong associations were also found in partially adjusted models which included background variables and all mental ill health variables (but not CP). However, in the fully adjusted model, there was only moderate evidence of an association for PTSD OR = 1.98 (95% CI 1.19–3.32); weak evidence for depression OR = 1.76 (95% CI 1.05–2.96); and no evidence for anxiety OR = 1.49 (95% CI 0.92–2.41). There was very strong evidence in all models, that both moderate CP (OR = 3.80, 95% CI 1.84–7.86) and severe CP (OR = 19.1, 95% CI 9.22–39.5) were associated with poor PGH. Gender did not modify the associations of mental health or CP with PGH.

**Table 4:** Crude and adjusted logistic regression models of poor perceived general health^a^

		Unadjusted		Partially adjusted^c^		Partially adjusted^c^		Fully adjusted^c^		
		Model 1		Model 2		Model 3		Model 4		
		uOR	95% CI	aOR	95% CI	aOR	95% CI	aOR	95% CI	Wald^b^
Poor perceived general health										
Gender^b^	Women	1.43^*^	(1.08–1.90)	1.64^*^	(1.11–2.41)			1.48	(0.97–2.28)	
HSCL-Anxiety^b^	Yes	4.81^***^	(3.54–6.54)	2.03^**^	(1.31–3.17)			1.49	(0.92–2.41)	0.241
HSCL-Depression^b^	Yes	5.21^***^	(3.89–6.97)	2.43^***^	(1.53–3.87)			1.76^*^	(1.05–2.96)	0.224
HTQ-PTSD^b^	Yes	5.18^***^	(3.80–7.06)	2.37^***^	(1.49–3.77)			1.98^**^	(1.19–3.32)	0.192
Chronic pain^b^	Some	5.70^***^	(2.83–11.5)			4.58^***^	(2.24–9.40)	3.80^***^	(1.84–7.86)	0.100
	Severe	42.9^***^	(21.4–86.1)			32.4^***^	(15.9–66.2)	19.1^***^	(9.22–39.5)	0.209

Reference groups: men (gender); no (mental health variables); no (somatic pain)

^a^ Perceived general health: fair/poor/very poor vs. good/very good

^b^ Interaction between gender and each of the three mental health variables were tested using Wald test of H_0_ = no interaction. Interactions were tested sequentially in adjusted models (i.e. one interaction term was tested at a time)

^c^ Adjusted models controlled for age, education, marital status, immigration year, PTE-AR and other variables estimated in the model

^*^p < 0.05

^**^p < 0.01

^***^p < 0.001

Table 5 shows functional impairment (FI) regressed on gender, mental ill health variables, CP and background variables (not shown in table) using logistic regression. Gender was not associated with FI in any model. Anxiety, depression and PTSD showed very strong associations with FI in crude models and fairly strong associations in partially adjusted models. In fully adjusted models, there was fairly strong evidence of interaction between gender and all mental ill health variables – i.e. the associations of anxiety, depression and PTSD with FI were gender specific. For women, there was no evidence of an association between any of the mental ill health variables and FI (Model 5: Women). In contrast, for men, there was very strong evidence that both anxiety and PTSD were associated with more than three times higher odds of FI. No association, however, was found between depression and FI in men. Severe CP was strongly associated with FI in all models, though moderate CP was not significantly associated with FI in the fully adjusted model. Finally, gender did not modify the association between CP and FI.

**Table 5:** Crude and adjusted logistic regression models of functional impairment^a^

		Unadjusted		Partially adjusted^c^		Partially adjusted^c^		Fully adjusted^c^		Gender-specific (fully adjusted^c^)				
		Model 1		Model 2		Model 3		Model 4		Model 5				
										Men		Women		
		uOR	95% CI	aOR	95% CI	aOR	95% CI	aOR	95% CI	aOR	95% CI	aOR	95% CI	Wald^b^
Functional impairment														
Gender^b^	Women	1.09	(0.81–1.48)	1.11	(0.73–1.69)			0.93	(0.59–1.48)	NA		NA		
HSCL-Anxiety^b^	Yes	6.24^***^	(4.48–8.70)	2.79^***^	(1.75–4.46)			2.25^**^	(1.31–3.87)	3.23^***^	(1.71–6.10)	1.21	(0.56–2.61)	0.027
HSCL-Dep^b^	Yes	5.70^***^	(4.15–7.85)	1.94^**^	(1.19–3.16)			1.25	(0.72–2.20)	1.83	(0.95–3.52)	0.66	(0.30–1.46)	0.024
HTQ-PTSD^b^	Yes	6.22^***^	(4.42–8.76)	2.54^***^	(1.56–4.13)			2.12^**^	(1.21–3.71)	3.12^***^	(1.62–6.02)	1.12	(0.51–2.43)	0.023
Chronic pain^b^	Moderate	3.88^**^	(1.62–9.3)			3.22^*^	(1.32–7.9)	2.50	(1.00–6.27)	2.50	(1.00–6.27)	2.50	(1.00–6.27)	0.385
	Severe	44.1^***^	(19.6–99.0)			35.6^***^	(15.5–81.5)	20.3^***^	(8.56–48.0)	20.3^***^	(8.56–48.0)	20.3^c^	(8.56–48.0)	0.704

Reference groups: men (gender); no (mental health variables); no (somatic pain)

^a^ Functional impairment (daily activities): some/a lot vs. no impairment

^b^ Interaction between gender and each of the three mental health variables were tested using Wald test of H_0_ = no interaction (ns = *p* > 0.10). Interactions were tested sequentially in adjusted models (i.e. one interaction term was tested at a time)

^c^ Adjusted models controlled for age, education, marital status, PTE-AR and other variables estimated in the model

^*^p < 0.05

^**^p < 0.01

^***^p < 0.001

## Discussion

### Summary of main findings

The present cross-sectional survey of adult refugees from Syria recently resettled in Norway is one of only a few studies investigating the burden of chronic pain (CP) and how it relates to mental ill health in a general refugee population. The study found high levels of CP and clear associations between CP and mental ill health (i.e. anxiety, depression and PTSD). Being a woman was associated with higher odds of CP and there was some evidence that gender acted as an effect modifier in the links between mental ill health and functional impairment (FI). Specifically, findings suggested a strong association of anxiety and PTSD with functional impairment in men, though not in women. Unsurprisingly, there was a strong independent positive association between age and chronic pain.

### The burden of chronic pain

The proportion reporting severe CP in the present sample, estimated at 43.1%, is moderately higher than that found by Strømme et al. in their studies on resettled adult refugees from Syria in Norway, where CP was reported by about 30% of participants both at baseline and follow-up one year later [27]. One possible explanation for this difference is that the study by Strømme et. al. measured CP with a single item whereas our measure was more comprehensive. Another possible explanation is the higher degree of exposure to potentially traumatic experiences (PTEs) and elevated levels of mental ill health in our sample compared with the sample in Strømme et. al. Our collaborating project in Sweden, with very similar study population, methodology and prevalence estimates of mental ill health [59, 60], however, also found that close to 30% of participating refugees experienced pain/discomfort at moderate/severe/extreme levels. Though, in the Swedish survey, pain was also measured by a single item from the standardized EQ-5D questionnaire developed by the EuroQol Group [69]. Furthermore, the EQ-5D item does not specify any body region and asks about pain today – i.e. the chronicity of pain is not investigated. Perhaps of some comparative value, another large study on Syrian refugees in Turkey found that almost 40% reported pain causing distress in both “arms, legs or joints” and “back” based the PHQ-15 [28]. However, the questionnaire-based level of anxiety differed slightly from our sample (34.7% vs. 30.1% respectively), and the levels of depression and PTSD were notably lower (36.5% vs. 45.2% and 19.6% vs. 29.7%, respectively). Our estimate for CP is considerably lower than estimates from studies on clinical or torture-exposed samples of refugees, which typically lie between 60 and 90% [21, 22, 24, 29, 70]. However, the proportion with severe CP among those reporting exposure to torture in our study (*n* = 231) was 57.8% (not shown in tables). As pain is a known long-term sequela of torture (e.g. [36]), higher levels of CP among torture-exposed refugees is unsurprising.

Interestingly and importantly, the percentage with severe musculoskeletal pain/complaints (i.e. the first five items of the CP measure) in our study was markedly higher than that found for the Norwegian general population using the “identical” measure: 35.2% vs 15.8% (see Additional file 2—distributions). Since one item, “pain in other regions”, was left out in our study, the true difference is likely even larger. Part of the reason for this notable difference may be related to poor transcultural construct validity of the instrument used to measure pain – i.e. “pain” may be understood and communicated differently in different cultures, including in Syrians [71, 72]. Another possible and related reason given local idioms of distress and social pressures against expressing negative feelings, is that the high level of pain reflects a more culturally acceptable expression of distress [12]. Alternatively, it represents a fairly accurate and transculturally valid approximated prevalence of chronic musculoskeletal pain as conceptualized and operationalized in the measure used in the present study. Given the known and strong links between pain and psychopathology (e.g. [4]) and the high levels of mental ill health in the study population as highlighted above, this is likely at least partially true. Furthermore, 38.0% of the sample reported having experienced physical violence, torture and/or sexual violence, which is known to be associated with subsequent pain [35, 36]. Additionally, during the civil war and during their flight to Norway, most refugees will not only have been without adequate access to health care and medicine but also, for many, adequate nourishment and rest. This might increase the risk of a variety of acute and chronic illnesses including musculoskeletal pain. Tying into a greater debate on how traumatic stress, and in particular chronic or continuous traumatic stress (CTS), is linked to pain in more resource poor settings, the high reported frequency of pain in our study may be a consequence of elevated levels of CTS in the study population [73]. That is, as stress caused by pre- and peri-migratory traumatic experiences is compounded by stress from a range of well-known adverse social conditions in the post-migratory environment such as financial hardship, unemployment, discrimination and weak social networks, the overall allostatic load may exceed coping capacities, with resultant consequences for health – e.g. elevated levels of pain [74, 75]. This resonates well with the currently dominating ecological/holistic approach to refugee health. This approach emphasizes the importance of investigating and addressing the full range of factors known to impact health: past trauma history, daily stressors and the disruption of psychosocial systems in the post-migration environment, and the interplay between them [18, 76]. A few recent studies have exploratively used biomarkers (e.g. hair cortisol levels) to assess stress/allostatic load when investigating mental ill health in refugee populations, with promising results [77, 78]. Given some of the inherent limitations of using self-report questionnaire data (e.g. information/recall bias), and that these limitations are likely exacerbated when working across languages, cultures and idioms of distress, the use of biomarkers could represent an interesting option for future research.

### The association between chronic pain and mental health

The strong association between mental ill health and CP in the present study is consistent with a large body of prior evidence from non-refugee populations [3–8] and a number of studies on torture-exposed refugees and refugees in treatment [24, 41, 43]. It is also consistent with a biopsychosocial approach to pain in which physical disorders such as pain is seen to result from a dynamic interaction among physiologic, psychological and social factors [79]. Very few prior studies on this topic exist on general refugee populations, adding to the uniqueness of the present study. The only study we are aware of, found relatively strong associations between CP and anxiety/depression and PTSD symptoms among resettled adult Syrian refugees in Norway [37]. If comparative evidence is broadened to also include studies on somatic distress, where pain is a key component, several recent, large studies on refugees and internally displaced people have documented strong links between symptoms of anxiety, depression and PTSD and somatic distress [28, 38, 39]. The clear reductions in the ORs when going from crude to fully adjusted models for all mental ill health variables in Table 3, suggests the associations between these variables and CP are overlapping, which is unsurprising given their known comorbidities. Nonetheless, each mental ill health variable was statistically significantly associated with CP in the fully adjusted model, suggesting a unique association above and beyond shared ones. Methodologically, comparing ORs across models with different predictors is not unproblematic due to scaling effects [80], thus this argument needs to be interpreted with caution. Our finding that being a woman was associated with increased risk of CP is consistent with prior evidence both in refugee (e.g. [28, 38]) and non-refugee populations (e.g. [45, 46]).

### Perceived general health and functional impairment

The mean value for perceived general health (PGH) in this study (3.614, SD = 1.063) was statistically significantly lower (though not necessarily clinically meaningfully lower) than that reported in a large study on general, adult (> 15 years), populations across 26 European countries using the identical instrument (*p* < 0.001), where the mean value was 3.724 (SD = 0.968) [81]. Since refugees in our study were significantly younger that in the European study (mean age 38.9 vs. 48.5 years), and given the strong negative association between age and PGH, the age-standardized difference is likely notably larger. The proportion in our study who reported “fair/poor/very poor” PGH was 39.9%, which is slightly higher than that reported in a longitudinal study on resettled refugees (primarily of Middle eastern origin) in Australia, which found this percentage to be 35.7% at baseline [82]. However, the scoring scale for PGH had six levels vs. five in our study. An interesting finding from the regression analyses on PGH was the weak evidence of association between the mental ill health variables and PGH after controlling for CP. This implies a high degree of overlap between the associations of mental ill health and CP with PGH, even if a moderately strong unique association was found between PTSD and PGH in the fully adjusted model. The strong association between CP and PGH is broadly in line with a study on female Yazidi refugees exposed to violence by the “Islamic State”, which found pain to be a strong predictor of overall health-related well-being [32].

The proportion of refugees who reported at least some degree of functional impairment (FI) due to longstanding illness in our study (34.9%) is fairly consistent with another study on resettled adult refugees from Syria in Norway, which found this proportion to be around 30%, both at baseline and one year later [27]. A similar estimate (35.8%) was also found in the aforementioned Australian study [82], though the measurement used also included long term injury/health conditions (i.e. the focus was not on FI per se). Our finding that CP was strongly associated with FI in the fully adjusted model which included mental ill health, is broadly congruent with other studies on refugees [38, 43], though these studies investigated somatic distress and not CP. One important and hitherto unique finding in our study was that the associations between mental ill health and FI were highly gender specific, with a clear association in men, but none in women in fully adjusted models. We are unable to relate this directly to existing literature, though prior studies have highlighted the importance of taking a gendered perspective when exploring refugee mental health [48–50]. Part of the explanation for this gender specificity may relate to the aforementioned tendency for mental ill health, or, specifically anxiety, to be more strongly linked to CP in women than men in our study. That is, when FI is regressed on both CP and mental ill health for women, any adverse associations of mental ill health may be too overlapping with that of CP to show a unique association. However, the tendency for gender-specific associations was also present in the partially adjusted models (without CP), even if the statistical evidence from Wald test of interaction was weaker. Another possible explanation is that the gendered association may be related to differences in expected and actual daily tasks and demands for men and women given traditional gender roles in Arab culture, with the identity of men more closely tied to work and being able to provide for their families [83–85]. With a relatively low percentage of paid employment among participants based on self-reported data (35% and 12% for men and women, respectively), it could be that unemployment was a driver of both mental ill health and a sense of impaired functioning especially in males given the centrality of work to men’s gender role in Arab culture. Alternatively, with almost threefold as many men as women working in the sample, it could be that symptoms of mental ill health were perceived as more impairing to functioning for a higher proportion of men (that is, assuming the link between mental ill health and function impairment was felt in particular in the context of work). Both of these explanations are exploratory in nature, though, and need to be investigated in future studies.

### Strengths and limitations

A key and potentially serious limitation with the present study is selection bias. With a response rate of 10%, it is hard to gauge the generalizability of findings. In general, predictor-outcome associations are thought to be more robust to nonresponse than prevalence estimates [86], though we cannot exclude that selection bias has distorted results. In prior publications, we have shown that the proportion of young and unmarried refugees was notably smaller among participants compared to the source population, though there was no difference in the proportion of women [68]. Furthermore, there was no difference in the proportion with anxiety and depression scores above cut-off when participants who returned the questionnaire within a month after study launch (*n* = 433) were compared with those who responded later and after a reminder had been sent out (*n* = 464). There was a tendency, however, that a higher proportion of early-responders had a PTSD score above cut-off [60]. This provides some evidence that willingness to participate (operationalized through how soon participants returned the questionnaire) was not strongly related to mental ill health. Another limitation concerns the validity of the instruments used in a population of adult refugees from Syria. We have already discussed transcultural construct validity relating to how pain was measured, and the issue of validity is also relevant for the HSCL-25 and HTQ scales, even if both scales have been used extensively with refugee populations and psychometrically tested. Studies are somewhat incongruent as to the cultural appropriateness and validity of the scales across settings and populations [63, 87], with recent evidence suggesting the depression subscale of the HSCL may have poor psychometric properties among Arabic speaking refugees and that the inclusion of purely somatic symptoms in the anxiety subscale may be inappropriate [64]. Moreover, only five items of the 10-item measure for chronic pain were from a previously used scale [53], with the rest composed for the purpose of the present study. As such, the psychometric properties of the full 10-item scale have not been tested. A further limitation concerns the cross-sectional design of the data, which prohibits causal interpretations. It is highly likely that CP and mental ill health are causally related, thus when placing both in the same regression model, there is a chance of overadjustment bias [88]. For example, if anxiety leads to elevated levels of perceived pain because of shared physiological pathways [89], part of the “true” adverse effect of anxiety on PGH may be masked by overadjustment. Moreover, the association between anxiety and FI in men could be because men with FI get anxious if they cannot fulfil their role as breadwinner, rather the other way around. Lastly, since regression models were built with data at hand, without detailed pre-registered plans for data-handling and analyses, findings should be viewed as partly exploratory, with the associated risk of false-positive findings [52, 90].

Strengths of the study include the random sampling from total-population registries; the fairly thorough assessment of CP compared to available evidence, and the use of well-known and frequently used instruments to measure mental ill health (despite the above noted lingering issues of transcultural validity).

## Conclusions

The present study shows a high burden of chronic pain in a general population of adult refugees from Syria with likely substantial adverse consequences for their daily functioning. The clear links found between chronic pain and mental ill health suggest that healthcare providers and others working with this population ought to be aware of, and sensitized to, the fact that pain often goes hand-in-hand with psychological strain (indeed, within the biopsychosocial framework, mental and physical health closely interact and share physiologic pathways and feedback loops, blurring the conceptual boundaries between them). This awareness may be particularly important with Syrian refugees given that illness models and expressions of distress of most Syrians do not clearly distinguish between the mental and physical and the cultural stigma against expressing psychological distress. Addressing the possible co-occurrence of pain and mental ill health may be required for interventions to effectively address either problem. The study further points to potential gender-specific associations between mental ill health and functional impairment, with associations seemingly stronger in men than in women, suggesting initiatives addressing mental ill health, chronic pain or functional impairment in refugee populations should consider gender in tailoring their content and outreach. The present study’s low response rate, despite substantial promotional work to boost participation [68], suggests that distributing these types of surveys via postal mail might not be the best strategy to recruit participants. Future studies may want to consider alternative designs (e.g. designs allowing for more face-to-face interaction with potential participants in order to thoroughly explain the purpose and rational of the study in the hope that this may increase participation).

A clear mismatch exists between the burden on health caused by pain in general refugee populations and the amount of available evidence to guide mitigating strategies. The fairly scant number of existing studies are characterized by marked methodological heterogeneity making comparison challenging and knowledge difficult to synthesize. Therefore, future research should aim towards harmonizing and standardizing methodologies for how pain is measured. Moreover, since pain is understood differently across cultures, it will be important for any such effort to take into consideration that pain is an ethnocultural construct in order to enhance transcultural validity.

## Supplementary Information

**Additional file 1.**

**Additional file 2: a.** Detailed distributions for musculoskeletal pain/stiffness and general pain. **b.** Fully adjusted ordered logistic regression models of Musculoskeletal pain/stiffness and General pain (i.e. compare with Table 3 in main manuscript).

**Additional file 3:** Sensitivity analyses of all fully adjusted regression models (i.e. last models in Tables 3, 4 and 5) with anxiety, depression and PTSD combined into one new variable “Any Mental Health Problem” (0 if *all* of anxiety/depression/PTSD =”No” and 1 if *any* of anxiety/depression/PTSD=”Yes”).

## Data Availability

The datasets generated and/or analyzed during the current study are not publicly available due to agreements made with participants through the informed consent form and outlined procedures for data-handling therein. Anonymized data are available from the corresponding author on reasonable request.

## Abbreviations

CI: Confidence Intervals
CP: Chronic Pain
DSM: Diagnostic and Statistical Manual of Mental Disorders
FI: Functional Impairment
HSCL: Hopkins Symptom Checklist
HTQ: Harvard Trauma Questionnaire
ICD: International Classification of Diseases
PGH: Perceived General Health
PHQ: Patient Health Questionnaire
PTSD: Post-traumatic Stress Disorder
PTE: Potentially Traumatic Experiences
PTE-AR: Potentially Traumatic Experiences Adversity Ratio
RTHC: Refugee Trauma History Checklist
SD: Standard Deviation
