# Supplementary material for: Chronic pain, mental health and functional impairment in adult refugees from Syria resettled in Norway: a cross-sectional study
Source: BMC Psychiatry. 2022 Aug 24;22:571. doi: 10.1186/s12888-022-04200-x (PMC9404590; doi:10.1186/s12888-022-04200-x)
Supplement: Supplementary file 1 — Additional file 1. [file 12888_2022_4200_MOESM1_ESM.docx]

**Additional file 1**

Main deviations from the pre-registered protocol (PRP), which can be found below, are:

- The chronic pain (CP) variable in the PRP was split into two components: pain in muscle and joints, and general pain. In the submitted manuscript, main tables/analyses combined these two pain variables into *one, overall chronic* *pain* variable. Frequency distributions if the pain variable was split are included as Additional file 2

- In the PRP, sleep difficulties/insomnia was to be included as an outcome in the present article. However, in order to have sufficient space to go into relevant detail, we decided to write a separate article for sleep difficulties/insomnia. This has been submitted to a different journal.

- In the present manuscript, *Impaired functioning* was dichotomized whereas in the PRP the outlined plan was to present descriptives for all three outcome categories: no/some/a lot (the manuscript combined the latter two categories into one)

- None of the regression analyses were pre-planned/registered, thus these analyses must be interpreted as exploratory rather than true hypothesis-testing as pointed out in the main manuscript.

Original registration in ClinicalTrials.com (italics indicate directly copied from registration)

Description of how key variables would be handled:

*Somatic pain: Likert scale [ Time Frame: Collected as part of the survey questionnaire sent out to the sample of participants. Participants will have 6 weeks to answer and return the survey. Anticipated time frame for collection: November 27, 2018 to January 15, 2019. ]*

*The questions on pain are based on The Tromsø Study, but slightly adapted to the study population of REFUGE-I (Andorsen, Ahmed, Emaus, & Klouman, 2016; "The Tromsø Study," n.d.). Participants will be asked about pain in muscles and joints in the last year, lasting for a minimum of 3 months, scored on a 3-point Likert scale (Not troubled; Somewhat troubled; and Very troubled) in the following 5 areas: Neck and shoulders; Arms or hands; Upper back, Lower back; and Hips, legs or feet. Similarly, using the same 3-point scale, participants will be asked about general pain in the following 5 areas: Stomach; Head; Genital area; Chest; and Other. The translation of the questions into Arabic was done by a professional translation bureau, then checked by two independent translators/interpreters. In addition, a thorough review was conducted by an Arabic speaking reference group from Syria.*

*Perceived general health [ Time Frame: Collected as part of the survey questionnaire sent out to the sample of participants. Participants will have 6 weeks to answer and return the survey. Anticipated time frame for collection: November 27, 2018 to January 15, 2019. ]*

*Two questions will used to measure perceived general health. Both questions are from the European Social Survey. Question one on overall health, "How is your health in general", has six answer categories: Very good; Good; Fair; Bad; Very bad; and Don't know. Question two, "Are you hampered in your daily activities in any way by any longstanding illness, or disability, infirmity or mental health problem?", taps into functional impairment and has four answer categories: No; Yes to some extent; Yes a lot; Don't know. The translation of the questions into Arabic was done by a professional translation bureau, then checked by two independent translators/interpreters. In addition, a thorough review was conducted by an Arabic speaking reference group from Syria.*

The section on cut-off for HTQ/HSCL

*Prevalence of symptom-based posttraumatic stress disorder (PTSD) as measured through the first 16 items in the section on trauma symptoms (section IV) in the Harvard Trauma Questionnaire (HTQ). A mean-item score ≥ 2.06 will define a "checklist-positive" PTSD case. To be included in analyses, participants must have answered 14 or more of the 16 items on the HTQ scale.*

*Prevalence of symptom-based depression and anxiety as measured through the two subscales on depression and anxiety in the Hopkins Symptom Checklist (HSCL-25). A mean-item score ≥ 1.80 and ≥ 1.75 will define a "checklist-positive" depression and anxiety case, respectively. To be included in analyses, participants must have answered ≥ 23 items on the 25-item scale.*

Planned manuscript/analyses:

*Title: "Subjective health and pain, and sleep difficulties in adult Syrian refugees resettled in Norway between 2015 and 2017: a cross-sectional survey questionnaire study".*

*Main outcomes:*

*Somatic pain (including pain in muscles and joints and general pain), measured as described under Outcome Measures. Statistics on pain in muscles and joints will be aggregated (i.e. all items will be combined) and reported as the percentage of participants in each of three categories: Not troubled with pain in muscles and joints (Not troubled reported for all items); Somewhat troubled with pain in muscles and joints (Somewhat troubled reported for at least one item); Very troubled with pain in muscles and joints (Very troubled for at least one item). Participants must have answered at least four of the five items (if all answers are Not troubled), or Somewhat troubled/Very troubled on at least one item to be included in analyses. Statistics on general pain will be presented in an identical manner (i.e. the percentage in each of three categories, aggregated across all items on general pain) Sleep difficulties, measured as described under Outcome Measures. The overall mean-item score will be reported, as well as the estimated prevalence of insomnia, defined by the following criteria: a score of 3 or above on at least one of the first 4 items + a score of 3 or higher on at least one of the last 2 items. Participants must have answered at least five of the six items to be included in analyses when analyzing the mean-item score of the scale. When analyzing the prevalence of insomnia, participants must have answered at least three of the first four items and one of the last two items; or, they must have a score of 3 or above on at least one of the first four items and a score of 3 or above on at least one of the last two items to be included in analyses.*

*Perceived general health, measured as described under Outcome Measures. Statistics on perceived general health will be reported as the percentage of participants in each answer category (with 95% CIs). Question one on overall health will be dichotomized with Very good/Good as one category and the remaining three as one category (Don't know will not be included). Question two on functional impairment will use all three answer categories (Don't know will not be included).*

*The outcome variables above (with 95% CIs) will be presented for the whole sample combined, and for the sample split on demographic and background variables 1-6 as outlined under the description of article I above. In addition, the outcome variables will be presented across the main outcome variables of article I: depression; anxiety; and PTSD (all binary). Furthermore, perceived general health will be reported across outcome variable one: pain in muscles and joints (3 categories) and general pain (3 categories); and outcome variable two: sleep difficulties (binary - insomnia vs. not). The number of participants contributing data to a specific statistics/analysis will be reported.*
