# Supplementary material for: Chronic pain, mental health and functional impairment in adult refugees from Syria resettled in Norway: a cross-sectional study
Source: BMC Psychiatry. 2022 Aug 24;22:571. doi: 10.1186/s12888-022-04200-x (PMC9404590; doi:10.1186/s12888-022-04200-x)
Supplement: Supplementary file 2 — Additional file 2: a. Detailed distributions for musculoskeletal pain/stiffness and general pain. b. Fully adjusted ordered logistic regression models of Musculoskeletal pain/stiffness and General pain (i.e. compare with Table 3 in main manuscript). [file 12888_2022_4200_MOESM2_ESM.docx]

| **Additional file 2a** Detailed distributions for musculoskeletal pain/stiffness and general pain | | | | | | | | | | | | | | | | | | | | |
| --- | --- | --- | --- | --- | --- | --- | --- | --- | --- | --- | --- | --- | --- | --- | --- | --- | --- | --- | --- | --- |
|  |  | Musculoskeletal pain/stiffness | | | | | | | | |  | General pain | | | | | | | | |
|  |  | No | |  | Some | |  | Severe | |  |  | No | |  | Some | |  | Severe | |  |
|  |  | n | (%) |  | n | (%) |  | n | (%) |  |  | n | (%) |  | n | (%) |  | n | (%) |  |
| Total |  | 255 | (28.8) |  | 318 | (35.9) |  | 313 | (35.2) |  |  | 311 | (35.5) |  | 296 | (33.7) |  | 270 | (30.8) |  |
| Gender | Men | 185 | (32.3) |  | 208 | (36.3) |  | 180 | (31.4) |  |  | 217 | (38.4) |  | 186 | (32.9) |  | 162 | (28.7) |  |
|  | Women | 70 | (22.4) |  | 110 | (35.1) |  | 133 | (42.5) |  |  | 94 | (30.1) |  | 110 | (35.3) |  | 108 | (34.6) |  |
| Age | 18 - 29 yrs | 90 | (46.6) |  | 67 | (34.7) |  | 36 | (18.7) |  |  | 86 | (45.3) |  | 54 | (28.4) |  | 50 | (26.3) |  |
|  | 30 - 39 yrs | 97 | (31.8) |  | 113 | (37.0) |  | 95 | (31.1) |  |  | 122 | (40.4) |  | 107 | (35.4) |  | 73 | (24.2) |  |
|  | 40 - 49 yrs | 48 | (21.1) |  | 91 | (39.9) |  | 89 | (39.0) |  |  | 78 | (34.5) |  | 70 | (31.0) |  | 78 | (34.5) |  |
|  | ≥50 | 20 | (12.5) |  | 47 | (29.4) |  | 93 | (58.1) |  |  | 25 | (15.7) |  | 65 | (40.9) |  | 69 | (43.4) |  |
| Education | ≤9 yrs | 96 | (24.7) |  | 146 | (37.6) |  | 146 | (37.6) |  |  | 119 | (31.1) |  | 131 | (34.2) |  | 133 | (34.7) |  |
|  | 10-12 yrs | 48 | (31.0) |  | 51 | (32.9) |  | 56 | (36.1) |  |  | 58 | (38.2) |  | 49 | (32.2) |  | 45 | (29.6) |  |
|  | >12 yrs | 103 | (31.6) |  | 119 | (36.5) |  | 104 | (31.9) |  |  | 127 | (39.4) |  | 109 | (33.9) |  | 86 | (26.7) |  |
| Marital status | Unmarried | 98 | (42.4) |  | 79 | (34.2) |  | 54 | (23.4) |  |  | 93 | (41.0) |  | 75 | (33.0) |  | 59 | (26.0) |  |
|  | Mar./partner | 152 | (25.9) |  | 214 | (36.5) |  | 221 | (37.6) |  |  | 207 | (35.5) |  | 199 | (34.1) |  | 177 | (30.4) |  |
|  | Divorced/wid. | 5 | (7.4) |  | 25 | (36.8) |  | 38 | (55.9) |  |  | 11 | (16.4) |  | 22 | (32.8) |  | 34 | (50.7) |  |
| Ref. status | Asylum seeker | 138 | (30.7) |  | 173 | (38.5) |  | 138 | (30.7) |  |  | 173 | (39.1) |  | 149 | (33.7) |  | 120 | (27.1) |  |
|  | Quota refugee | 66 | (25.0) |  | 88 | (33.3) |  | 110 | (41.7) |  |  | 78 | (29.8) |  | 95 | (36.3) |  | 89 | (34.0) |  |
|  | Family reunion | 38 | (28.6) |  | 47 | (35.3) |  | 48 | (36.1) |  |  | 50 | (37.9) |  | 40 | (30.3) |  | 42 | (31.8) |  |
| Immigr. year | 2010-2015 | 152 | (30.3) |  | 184 | (36.7) |  | 165 | (32.9) |  |  | 188 | (37.9) |  | 160 | (32.3) |  | 148 | (29.8) |  |
|  | 2016 | 39 | (24.4) |  | 44 | (27.5) |  | 77 | (48.1) |  |  | 46 | (28.8) |  | 55 | (34.4) |  | 59 | (36.9) |  |
|  | 2017 | 51 | (28.3) |  | 70 | (38.9) |  | 59 | (32.8) |  |  | 61 | (34.5) |  | 65 | (36.7) |  | 51 | (28.8) |  |
| PTE-AR | < 0.20 | 69 | (43.4) |  | 53 | (33.3) |  | 37 | (23.3) |  |  | 75 | (46.9) |  | 51 | (31.9) |  | 34 | (21.3) |  |
|  | 0.20-0.29 | 30 | (38.5) |  | 25 | (32.1) |  | 23 | (29.5) |  |  | 31 | (40.3) |  | 23 | (29.9) |  | 23 | (29.9) |  |
|  | 0.30-0.39 | 41 | (26.1) |  | 62 | (39.5) |  | 54 | (34.4) |  |  | 62 | (40.0) |  | 52 | (33.5) |  | 41 | (26.5) |  |
|  | ≥0.40 | 91 | (21.9) |  | 152 | (36.6) |  | 172 | (41.4) |  |  | 115 | (28.1) |  | 146 | (35.7) |  | 148 | (36.2) |  |
| HSCL-anx | No | 219 | (36.7) |  | 234 | (39.3) |  | 143 | (24.0) |  |  | 270 | (45.7) |  | 206 | (34.9) |  | 115 | (19.5) |  |
|  | Yes | 33 | (11.9) |  | 82 | (29.5) |  | 163 | (58.6) |  |  | 40 | (14.5) |  | 89 | (32.2) |  | 147 | (53.3) |  |
| HSCL-dep | No | 199 | (38.5) |  | 210 | (40.6) |  | 108 | (20.9) |  |  | 248 | (48.1) |  | 180 | (34.9) |  | 88 | (17.1) |  |
|  | Yes | 52 | (14.9) |  | 102 | (29.3) |  | 194 | (55.7) |  |  | 60 | (17.5) |  | 110 | (32.1) |  | 173 | (50.4) |  |
| HTQ-PTSD | No | 212 | (37.7) |  | 222 | (39.4) |  | 129 | (22.9) |  |  | 259 | (45.9) |  | 193 | (34.2) |  | 112 | (19.9) |  |
|  | Yes | 39 | (13.0) |  | 90 | (29.9) |  | 172 | (57.1) |  |  | 47 | (16.0) |  | 99 | (33.7) |  | 148 | (50.3) |  |

| **Additional file 2b** Fully adjusted ordered logistic regression models of Musculoskeletal pain/stiffness and General pain (i.e. compare with Table 3 in main manuscript) | | | | | | | | | | | |
| --- | --- | --- | --- | --- | --- | --- | --- | --- | --- | --- | --- |
|  |  |  | **Musculoskeletal pain/stiffness** | | | |  | **General pain** | | | |
|  |  |  | aOR | 95% CI | p-value | Wald^‡^ |  | aOR | 95% CI | p-value | Wald^‡^ |
| Gender‡ | Women |  | 1.73 | (1.27-2.36) | <0.001 |  |  | 1.29 | (0.96-1.74) | 0.096 |  |
| HSCL-Anxiety^‡^ | Yes |  | 2.17 | (1.50-3.14) | <0.001 | 0.534 |  | 2.31 | (1.61-3.30) | <0.001 | 0.057 |
| HSCL-Dep^‡^ | Yes |  | 2.04 | (1.38-3.04) | <0.001 | 0.244 |  | 2.22 | (1.52-3.26) | <0.001 | 0.163 |
| HTQ-PTSD^‡^ | Yes |  | 1.79 | (1.20-2.66) | 0.004 | 0.582 |  | 1.55 | (1.07-2.24) | 0.021 | 0.667 |
| Age | 30-39 years |  | 1.94 | (1.32-2.85) | 0.001 |  |  | 1.15 | (0.78-1.70) | 0.474 |  |
|  | 40-49 years |  | 2.88 | (1.87-4.43) | <0.001 |  |  | 1.64 | (1.06-2.53) | 0.027 |  |
|  | ≥50 years |  | 4.90 | (2.98-8.04) | <0.001 |  |  | 2.62 | (1.63-4.23) | <0.001 |  |
| Marital status | Married/partner |  | 1.35 | (0.93-1.95) | 0.112 |  |  | 0.98 | (0.68-1.42) | 0.933 |  |
|  | Prev. married§ |  | 2.45 | (1.33-4.49) | 0.004 |  |  | 1.83 | (1.00-3.32) | 0.048 |  |
| Immigration year | 2016/2017 |  | 1.17 | (0.87-1.56) | 0.302 |  |  | 1.32 | (0.98-1.76) | 0.064 |  |
| PTE-AR | 0.20-0.39 |  | 1.53 | (1.04-2.24) | 0.031 |  |  | 1.09 | (0.74-1.61) | 0.650 |  |
|  | ≥0.40 |  | 1.46 | (1.01-2.10) | 0.042 |  |  | 1.19 | (0.82-1.73) | 0.350 |  |
| Reference groups: men (gender); no (mental health variables); 18-29 years (age); unmarried (marital status); 2010-2015 (Immigration. year); <0.20 (PTE-AR)  ^‡^ Interaction between gender and each of the three mental health variables was tested using Wald test of H0=no interaction (ns=p>0.10). Interactions were tested sequentially – i.e., only one interaction term was tested at a time.  ^§^ Category included: divorced/separated/widow(er) | | | | | | | | | | | |
