# Supplementary material for: Chronic pain, mental health and functional impairment in adult refugees from Syria resettled in Norway: a cross-sectional study
Source: BMC Psychiatry. 2022 Aug 24;22:571. doi: 10.1186/s12888-022-04200-x (PMC9404590; doi:10.1186/s12888-022-04200-x)
Supplement: Supplementary file 3 — Additional file 3: Sensitivity analyses of all fully adjusted regression models (i.e. last models in Tables 3, 4 and 5) with anxiety, depression and PTSD combined into one new variable “Any Mental Health Problem” (0 if all of anxiety/depression/PTSD =”No” and 1 if any of anxiety/depression/PTSD=”Yes”). [file 12888_2022_4200_MOESM3_ESM.docx]

**Additional file 3.** Sensitivity analyses of all fully adjusted regression models (i.e. last models in Tables 3-5) with anxiety, depression and PTSD combined into one new variable “Any Mental Health Problem” (0 if *all* of anxiety/depression/PTSD =”No” and 1 if *any* of anxiety/depression/PTSD=”Yes”)

| 3a Ordered logistic regression of *chronic pain* (i.e. compare to Table 3 in main manuscript) | | | | | |
| --- | --- | --- | --- | --- | --- |
|  |  |  | aOR† | 95% CI | Wald‡ |
| Gender‡ | Women |  | 1.57b | (1.15-2.13) |  |
| Any Mental Health Problem | Yes |  | 5.87c | (4.34-7.94) | 0.489 |
| † Adjusted for age, marital status, immigration year and PTE-AR (in addition to gender and “Any mental health problem”)  ‡ Interaction between gender and “Any mental health problem” was tested using Wald test of H_0_=no interaction | | | | | |

| 3c Logistic regression of *functional impairment* (i.e. compare to Table 5 in main manuscript) | | | | | | | | | | | | |
| --- | --- | --- | --- | --- | --- | --- | --- | --- | --- | --- | --- | --- |
|  |  |  | Total | |  | Men | |  | Women | |  |  |
|  |  |  | aOR† | 95% CI |  | aOR† | 95% CI |  | aOR† | 95% CI |  | Wald‡ |
| Gender‡ | Women |  | 0.98 | (0.63-1.53) |  |  |  |  |  |  |  |  |
| Any Mental Health Problem | Yes |  | 3.37c | (2.20-5.15) |  | 4.77c | (2.78-8.18) |  | 1.82 | (0.91-3.60) |  | *0.027* |
| Chronic pain (CP) | Moderate |  | 2.77a | (1.14-6.71) |  | 2.77a | (1.14-6.71) |  | 2.77a | (1.14-6.71) |  | 0.431 |
|  | Severe |  | 22.8c | (9.58-54.2) |  | 22.8c | (9.58-54.2) |  | 22.8c | (9.58-54.2) |  | 0.630 |
| † Adjusted for age, marital status, immigration year and PTE-AR (in addition to gender, “Any mental health Problem” and CP)  ‡ Interactions between gender and “Any mental health problem” and CP were tested using Wald test of H_0_=no interaction. Since the interaction gender*CP was non-significant at the 0.1 threshold, aORs for men and women are set as identical in stratified model (even if they varied slightly) | | | | | | | | | | | | |

| 3b Logistic regression of *poor perceived general health* (i.e. compare to Table 4 in main manuscript) | | | | | | | | | | | | |
| --- | --- | --- | --- | --- | --- | --- | --- | --- | --- | --- | --- | --- |
|  |  |  | Total | |  | Men | |  | Women | |  |  |
|  |  |  | aOR† | 95% CI |  | aOR† | 95% CI |  | aOR† | 95% CI |  | Wald‡ |
| Gender‡ | Women |  | 1.52 | (0.99-2.34) |  |  |  |  |  |  |  |  |
| Any Mental Health Problem | Yes |  | 2.87c | (1.95-4.23) |  | 4.20 c | (2.58-6.83) |  | 1.51 | (0.83-2.77) |  | *0.007* |
| Chronic pain (CP) | Moderate |  | 3.97c | (1.88-3.55) |  | 3.97c | (1.88-3.55) |  | 3.97c | (1.88-3.55) |  | 0.112 |
|  | Severe |  | 21.6c | (10.3-45.6) |  | 21.6c | (10.3-45.6) |  | 21.6c | (10.3-45.6) |  | 0.200 |
| † Adjusted for age, marital status, immigration year and PTE-AR (in addition to gender, “Any mental health Problem” and CP)  ‡ Interactions between gender and “Any mental health problem” and CP were tested using Wald test of H_0_=no interaction. Since the interaction gender*CP was non-significant at the 0.1 threshold, aORs for men and women are set as identical in stratified model (even if they varied slightly) | | | | | | | | | | | | |
